# Psychosocial Vulnerabilities to Upper Respiratory Infectious Illness: Implications for Susceptibility to Coronavirus Disease 2019 (COVID-19)

**DOI:** 10.1177/1745691620942516

**Published:** 2020-07-08

**Authors:** Sheldon Cohen

**Affiliations:** Department of Psychology, Carnegie Mellon University

**Keywords:** health practices, psychological stress, social support, social integration, common cold, influenza, COVID-19

## Abstract

For 35 years, our laboratory has been involved in identifying psychosocial factors that predict who becomes ill when they are exposed to a virus affecting the upper respiratory tract. To pursue this question, we used a unique viral-challenge design in which we assessed behavioral, social, and psychological factors in healthy adults. We subsequently exposed these adults to a cold or influenza virus and then monitored them in quarantine for 5 to 6 days for onset of respiratory illness. Factors we found to be associated with greater risk of respiratory illnesses after virus exposure included smoking, ingesting an inadequate level of vitamin C, and chronic psychological stress. Those associated with decreased risk included social integration, social support, physical activity, adequate and efficient sleep, and moderate alcohol intake. We cautiously suggest that our findings could have implications for identifying who becomes ill when exposed to severe acute respiratory syndrome coronavirus 2 (SARS-CoV-2), the virus responsible for coronavirus disease 2019 (COVID-19). This argument is based on evidence that the associations we report are replicable across multiple respiratory viruses and that the pathways found to link psychosocial factors to colds and influenza may play similar roles in COVID-19.

For 35 years, our laboratory has studied the role of psychosocial factors in respiratory infectious diseases, including the common cold and influenza (https://www.commoncoldproject.com). Our primary aim has been to identify factors that predict who becomes ill when they are exposed to a virus. To pursue this question, we used a unique viral-challenge design in our studies. Volunteers screened for “good health” were assessed for a psychosocial variable of interest (e.g., smoking status, alcohol consumption, psychological stress, social support) and subsequently exposed to a virus (cold or influenza) that caused a mild upper respiratory illness. They were monitored in quarantine for the development of infection and symptoms. Across studies, 70% to 85% of participants exposed to a virus were infected, and 25% to 40% developed a verified upper respiratory disease.

In this article, I describe our research on three of the factors that we have studied as potential predictors of susceptibility to upper respiratory disease using the viral-challenge paradigm—risky behaviors, psychological stress, and social relationships. In each case, I include evidence for behavioral and physiological pathways that may account for associations we have detected. My intent in summarizing this work is to inform both the public and scientific community of the importance of behavior, psychological states, and social interactions to our health and, in particular, to the onset and progression of respiratory infectious diseases.

Of special importance in the midst of this pandemic is the possibility that our work with the common cold and influenza viruses may suggest predictors of susceptibility to Coronavirus Disease 2019 (COVID-2019). There has been much discussion regarding the behavioral determinants of exposure to severe acute respiratory syndrome coronavirus 2 (SARS-CoV-2), the cause of COVID-19. The application of social distancing, washing hands, cleaning surfaces touched often, and wearing gloves and masks are all examples of behaviors that reduce exposure to the virus (Jefferson et al., 2011). As mentioned earlier, our work on susceptibility to respiratory viruses focused on a different issue. What are the determinants of infection and illness among those who are exposed? Although understanding the determinants of exposure to SARS-CoV-2 has played a key role in response to the current pandemic, it is also essential to identify the factors that predict whether those exposed to SARS-CoV-2 become infected, develop COVID-19, and show disease progression and mortality (del Rio & Malani, 2020).

Very little is known right now about COVID-19 and even less is known about the potential role psychosocial factors play in risk for disease and death in persons exposed to SARS-CoV-2. But we can make educated guesses on the basis of evidence from other respiratory viruses. I argue that the potential generality of these results to COVID-19 is based on evidence that the associations we found are replicable across multiple respiratory viruses and that the pathways found to link psychosocial factors to colds and influenza may play similar roles in COVID-19. However, to be clear, I do not think that the results of this research on the risk factors for infection and illness in response to exposure to common cold and influenza viruses can be assumed to generalize to COVID-19. I propose only that they suggest profitable areas for scientific investigation.

I begin with a description of the methods used in our work on psychosocial factors as predictors of susceptibility to common cold and influenza viruses. I then provide summaries of our studies investigating the potential roles of risky behaviors, psychological stress, and interpersonal relationships in disease susceptibility. Each summary is followed by a discussion of the potential implications for identifying risk for COVID-19.

## Description of the Viral-Challenge Studies

### Participants

Volunteers in our studies ranged from 18 to 55 years old and qualified for participation if they were in “good general health” as determined by medical history, physical examination, and clinical profiles via urinalysis, complete blood cell count, and analysis of blood chemistry. They also could not (a) be taking any medications, (b) be seropositive for human immunodeficiency virus, (c) be pregnant, (d) have had an upper respiratory illness during the 30 days before exposure to the study virus, or (e) report to quarantine with any symptoms.

Across studies, the median age of our participants ranged from 29 to 35 years. A typical study had approximately equal numbers of men and women, and about 30% were married or in a marital-type relationship. The vast majority of participants were high school graduates; 75% had 2 or more years of college. Approximately 65% were White, 30% were Black, and 5% were of other racial/ethnic backgrounds. More than 60% were currently employed.

### Quarantine

Our early work (Cohen, Tyrrell, Russell, Jarvis, & Smith, 1993; Cohen, Tyrrell, & Smith, 1991) was conducted at the British Common Cold Unit (CCU) in Salisbury, England (Tyrrell & Fielder, 2002). At the CCU, participants were housed in flats alone or with one or two other participants. They were asked to maintain social distance from their roommates and to wash their hands often. They were allowed to take walks on the grounds of the CCU, remaining at least 30 feet from others.

The remaining studies were all conducted in Pittsburgh. Here, each participant was quarantined alone in a hotel room on a floor accessible only to study staff and participants. They spent the vast majority of the time in their rooms (reading, watching television, watching movies, etc.) but were allowed into common spaces for acquiring food at meal times, for medical tests, and for occasional informal interaction with staff and participants. They were required to wash their hands often during these periods, to maximize their social distance, and to avoid any physical contact. At no time were they allowed in other participants’ rooms.

### Reimbursement

Participants in the study conducted at Britain’s CCU were reimbursed for their travel to the CCU and for meals during travel and incidentals. In the Pittsburgh studies, participants were reimbursed between $500 and $1,000 for their time, depending on the requirements of the study.

### Procedures

Table 1 depicts the temporal sequence of a typical viral-challenge study conducted in our laboratory. Preexisting immunity (antibody level) to the challenge virus, race, age, educational attainment (years), sex, body mass index (weight in kilograms divided by the square of height in meters), and season of the year were assessed at the medical screening and used as covariates in all the reported analyses. Virus type was also included as a covariate in studies in which more than one virus was used. During the remaining baseline period (between screening and exposure to the virus), qualified volunteers completed questionnaires, interviews, and biomarker assessments that provided measures of potential psychological, social, and behavioral predictors. After the baseline data were collected, we exposed participants to a virus through nasal drops. We then followed them in quarantine for 5 or 6 days (depending on the virus) to assess whether they developed a verified upper respiratory illness (cold or influenza).

**Table 1.** Temporal Sequence of a Typical Trial

2 months before quarantine
Eligibility screening
Blood sample for preexisting antibody to virus^a^
1–4 weeks before quarantine
Psychosocial questionnaires (Session 1) and interviews
Demographic questionnaire
6–14 daily assessments of risky behaviors & social interactions^b^
Biomarker assessments^b^
Quarantine Day 0
Psychosocial questionnaires (Session 2)
Baseline nasal secretions for virus culture
Baseline signs and symptoms of respiratory illness
End of Day 0
*Inoculation with virus*
Quarantine Days 1 through 5–6
Daily nasal secretions for virus culture
Daily signs and symptoms of respiratory illness
4 weeks after virus challenge
Postexposure blood sample for antibody to virus

a In some trials, preexisting antibody levels were assessed on Quarantine Day 0 before the viral challenge.

b Biomarker assessments were performed in select trials.

### Study outcomes

#### Infection

Infectious diseases result from the growth and action of microorganisms or parasites in the body. Infection is the multiplication of an invading microorganism. When an upper respiratory virus replicates, it can be found in nasal-secretion samples. In our studies, a participant was considered infected if we recovered the challenge virus in his or her nasal secretions on any of the postchallenge days (Gwaltney, Colonno, Hamparian, & Turner, 1988).

Antibodies are protein molecules that attach themselves to invading microorganisms and mark them for destruction or prevent them from infecting cells. Because the immune system responds to an infection by producing antibodies to the infectious agent, increases in virus-specific antibody levels after virus exposure provide an indirect marker of infection. Hence, participants were also considered infected if they had a at least a fourfold rise in virus-specific serum neutralizing antibody titer (preexposure to 28 days postexposure; Gwaltney et al., 1988).

#### Symptoms and signs of illness

Not everyone who is infected by a virus develops the symptoms and signs (objective markers) of illness. In our early work at the British CCU (Cohen et al., 1991, 1993), illness was determined by a diagnosis by a physician according to a standard list of symptoms assessed during daily examinations. Examples of the symptoms included runny nose, congestion, cough, sore throat, malaise, sneezing, and nasal stuffiness. Physicians were blinded to all psychosocial data. Illness in the later (Pittsburgh) studies was assessed using two objective markers of upper respiratory illness: nasal mucus production and nasal mucociliary clearance function. Daily mucus production was assessed by collecting used tissues in sealed plastic bags (Doyle, McBride, Swarts, Hayden, & Gwaltney, 1988). Bags with soiled tissues were weighed and the weights of clean tissues and empty bags subtracted resulting in the weight of mucus produced. Clearance function refers to the effectiveness of nasal cilia (small hairs that move the mucus through the respiratory tract) in clearing mucus from the nasal passage toward the throat and is subjectively experienced as congestion. Clearance function was assessed as the time required for a saccharin-dyed solution administered into the nostrils to be tasted by the participant (Doyle et al., 1988). Here, illness was defined as (a) a total baseline-adjusted mucus weight of 10 g or more or (b) average baseline-adjusted nasal mucociliary clearance time of 7 min or longer (Cohen, Doyle, Skoner, Rabin, & Gwaltney, 1997).

#### Diagnosis of clinical disease

Participants were diagnosed as having a clinical disease only if they were both infected with the challenge virus and met the illness criteria. Changes in disease risk associated with psychosocial factors may occur because of changes in the rate of infection, in the rate of illness among infected persons, or both. Several of the studies discussed here included secondary analyses to determine whether one or both of these pathways were at play. Associations attributable to changes in infection rates suggest that psychosocial predictors may be influencing the early immune response (e.g., endothelial or lymphocyte production of interferon and natural killer cell activity), whereas those associated with changes in illness (expression of signs and symptoms) suggest processes involved in the production of symptoms (e.g., proinflammatory cytokine stimulation of inflammatory response).

All procedures, questionnaires, assay descriptions, data, and so forth for each of our studies are accessible via the Common Cold Project (CCP) website (https://www.commoncoldproject.com).

## Risky Behaviors and Disease Susceptibility

Behaviors such as smoking, excessive alcohol consumption, engaging in insufficient physical activity, not getting enough sleep, and eating poorly have been associated with incidence and progression of a range of diseases including cardiovascular disease, cancer, stroke, Type 2 diabetes mellitus, and chronic obstructive pulmonary disease (COPD; e.g., Ford et al., 2009) as well as with mortality (e.g., Mokdad, Marks, Stroup, & Gerberding, 2004). Many of these behaviors have also been found to correlate with the occurrence or severity of various infections; however, there has been little research on their potential role as risk factors for upper respiratory diseases.

### Risky behaviors, the cold, and influenza

In an early study (Cohen et al., 1993), we examined the relation of smoking and of alcohol consumption, assessed at baseline, with the risk for developing a cold in 391 participants who were subsequently exposed to one of five respiratory viruses (rhinovirus Types 2, 9, and 14, respiratory syncytial virus, or coronavirus 229E). Alcohol consumption was assessed with the use of a self-report questionnaire and smoking status with an objective indicator—serum levels of cotinine, a metabolite of nicotine. We found that smokers were at greater risk than nonsmokers for developing colds because smokers were more likely both to develop infections and to develop illness after infection. In contrast, greater numbers of alcoholic drinks (up to a maximum of 3 or 4 per day) were associated with decreased risk for developing colds because drinking was associated with decreased illness after infection (note that there were no heavy drinkers in our sample). However, only nonsmokers experienced the benefits of moderate drinking.

In a later study (Cohen et al., 1997), we examined 228 healthy volunteers exposed to one of two types of rhinovirus—RV-39 or Hanks—and assessed the independent associations of smoking, exercising, sleep efficiency (percentage of time sleeping while in bed), consuming alcohol, ingesting no more than 85 mg of vitamin C per day, and taking zinc supplements with the development of colds. In this study, all of the risky behaviors were assessed at baseline using questionnaires. After controlling for demographic variables and other potential confounders, we found that smokers were 3 times more likely to develop colds than nonsmokers. Those who exercised less often than twice a week were 1.8 times more likely to develop colds, and those with sleep efficiencies of less than 80% were 2.6 times more likely to develop a cold. In addition, moderate drinking (two to four drinks per day) again was associated with protection from the virus; those who did not drink regularly (consumed less than one alcoholic drink per day) were twice as likely to develop a cold. Finally, those who ingested no more than 85 mg of vitamin C per day were 2 times more likely to develop a cold. There were no associations between zinc and colds. The risky-behavior analyses were done simultaneously, so that each association reported above was independent of the other behaviors. The separate associations of these factors with infection and illness among the infected were not examined.

Over the past decade, better understanding of various components of sleep and the development of affordable and nonintrusive technologies to assess sleep in the natural environment led us to further pursue the role of sleep in susceptibility to upper respiratory infections. Recall that in the previous study (Cohen et al., 1997), using a sleep questionnaire at baseline, we found that those who slept less than 80% of their time in bed (sleep efficiencies of less than 80%) were 2.6 times more likely to develop a cold. In our first follow-up of this work (Cohen, Doyle, Alper, Janicki-Deverts, & Turner, 2009), we studied 153 healthy men and women who were interviewed about their sleep quality daily for 14 consecutive days before virus exposure, quarantined, administered nasal drops containing a rhinovirus (RV-39), and monitored for the development of a cold. Average scores for duration and sleep efficiency were calculated over the 14 days. Participants who averaged less than 7 hr of sleep were 2.94 times more likely to develop a cold than those who averaged 8 hr or more. Those with less than 92% efficiency were 5.50 times more likely to develop a cold than those with 98% or more efficiency.

In a subsequent study (Prather, Janicki-Deverts, Hall, & Cohen, 2015) of 164 men and women, we assessed sleep with actigraphs—wristwatch-like instruments that estimate sleep parameters on the basis of participants’ physical movements while they were in bed. Participants wore the actigraphs for 7 consecutive nights. After sleep assessments, they were exposed to RV-39 and quarantined. Here, again, shorter sleep duration was associated with an increased likelihood of the development of a clinical cold. Specifically, those sleeping less than 6 hr were at greater risk of developing the cold compared with those sleeping more than 7 hr per night. The association of sleep duration with cold incidence was primarily driven by illness expression among infected participants. Other sleep variables obtained using actigraphy were not strong predictors of cold susceptibility. The difference in minimum sleep requirements suggested by the earlier study (actigraphy about 6 hr and self-reported sleep about 7 hr) is consistent with differences in results for hours of sleep found when these two assessment methods are used simultaneously (e.g., Lauderdale, Knutson, Yan, Liu, & Rathouz, 2008).

### Implications for COVID-19

As mentioned earlier, behaviors we have studied are generally considered risk factors for a broad range of disease outcomes (Ford et al., 2009). This implies either that they influence some unitary process that is important for multiple diseases (e.g., immune suppression, inflammation), or that they influence multiple physiological systems and hence have implications for multiple diseases. Either explanation suggests their potential for playing a role in COVID-19.

Smoking is thought to increase the risk of respiratory infections by triggering inflammation or through structural changes in the respiratory tract or suppression of immune response (Zhou, Chen, & Peng, 2016). Our evidence from two of the studies of colds suggest broad effects of smoking, including increases in the risk for both infection and illness among the infected. Epidemiologic data indicate that cigarette smoking is also a substantial risk factor for influenza (Lawrence, Hunter, Murray, Lim, & McKeever, 2019). It would not be surprising if smoking increased risk and progression of COVID-19 through these same mechanisms. In fact, reviews of studies of data from the first few months of the COVID-19 pandemic (Guo, 2020; Vardavas & Nikitara, 2020) concluded that smoking is associated with negative progression and adverse outcomes of COVID-19. Likewise, a study from China found that smoking in men was associated with greater rates of disease (del Rio & Malani, 2020). However, the number of cases in most studies to date are low, and designs are often flawed (Berlin, Thomas, Le Faou, & Cornuz, 2020). More sophisticated studies with larger samples that control for alternative explanations and separate effects of exposure from those of host response to the virus should eventually provide a clearer picture of the association between smoking and COVID-19.

There is strong evidence that long-term alcohol abuse and acute binge drinking are associated with immunosuppression and increased susceptibility to both bacterial and viral infections (Barr, Helms, Grant, & Messaoudi, 2016; Sarkar, Jung, & Wang, 2015). However, increasing evidence suggests that small to moderate amounts of alcoholic beverages can be associated with an enhanced immune response (Romeo et al., 2007). This includes evidence that moderate drinkers have lower levels of circulating inflammatory markers (Ridker, Buring, Shih, Matias, & Hennekens, 1998).

In two studies, we found that those who abstain from consuming alcohol are at greater risk for colds than those who drink one to four drinks a day. Because we did not have heavier drinkers in our studies, we cannot conclude anything about the potential effects of consuming greater doses, although the evidence on alcohol abuse and immunity leads us to expect that greater numbers of drinks would be associated with greater risk (Barr et al., 2016; Messaoudi, Pasala, & Grant, 2014). A recent British study of risk for COVID-19 hospitalization similarly found greater risk among those who were nondrinkers before virus exposure but only weak evidence for an increase in risk for those who were heavier drinkers (Hamer, Kivimakib, Gale, & Batty, 2020). Associations between moderate drinking and more positive health have also been found in coronary heart disease and stroke (Emberson & Bennett, 2005). The reliability of our findings across seven of the upper respiratory viruses and the potential suppression of light to moderate drinking on the inflammatory response are consistent with a possible beneficial role of moderate drinking in COVID-19 (see Hamer et al., 2020). (Our analyses did not examine men and women separately, and moderate drinking for women is generally defined as about half that for men.) These data suggest that it would be worth investigating whether low to moderate rates of drinking are related to decreased risk of illness for COVID-19.

Although strong evidence has accumulated suggesting that better sleep enhances immune defenses (Besedovsky, Lange, & Born, 2012; Irwin, 2015), there is actually little direct evidence associating how long people sleep and their risk for infectious disease (Bryant & Curtis, 2013; Irwin, 2015). In our work, we found that both minimal sleep duration and low sleep efficiency predicted greater risk for colds. Secondary analyses suggested that the association of sleep duration with cold incidence was primarily driven by illness expression among infected participants. A likely pathway linking sleep to illness expression is an excessive inflammatory response (Irwin, 2015). It is noteworthy that current medical advice for preventing COVID-19 often includes the suggestion to get sufficient hours of sleep (e.g., Medalie, 2020; Mônico-Neto, dos Santos, & Antunes, 2020). Our work with cold and influenza viruses provides some empirical basis for this suggestion.

Prolonged, intense exercise suppresses the immune system, whereas moderate-intensity exercise improves immune function, potentially decreasing the risk and severity of respiratory viral infection by reducing excessive local inflammation (Martin, Pence, & Woods, 2009; Nieman, 1994). In the single study in which we investigated exercise, we found that those who exercised less than twice a week were 1.8 times more likely to develop a cold. That moderate exercise is associated with the reduction of local inflammation, together with the association between moderate exercise and a decreased risk for colds, suggests the possibility that exercise plays a similar role in risk for COVID-19. In fact, recent evidence found that relatively low levels of physical activity assessed before virus exposure protected against COVID-19 hospitalization (Hamer et al., 2020).

Vitamin C contributes to immune defense by supporting various cellular functions of both the innate and adaptive immune system (Carr & Maggini, 2017). We found that those ingesting no more than 85 mg of vitamin C per day were twice as likely to develop a cold. Our data vary from the broader literature on vitamin C and upper respiratory disease in that we assessed whether participants met a minimum level of vitamin C intake in their regular diets, as opposed to evaluating high-dose supplementation (≥ 200 mg). The supplementation research shows only small effects on cold incidence (Hemila & Chalker, 2013) and does not provide a convincing argument for the possible importance of high doses of vitamin C in COVID-19. Although it is based on only one study at this time, our work indicates that, by contrast, low (insufficient) dietary levels of vitamin C may play an important role.

## Psychological Stress and Disease Susceptibility

Psychological stress occurs when an individual perceives that environmental demands tax or exceed his or her adaptive capacity (Lazarus & Folkman, 1984). Operationally speaking, studies of psychological stress focus either on the occurrence of environmental events that are consensually judged as taxing or on individual responses to events that are indicative of this overload, such as perceived stress and event-elicited negative affect. Generally, stressful events are thought to influence the pathogenesis of physical disease by causing negative affective states (e.g., feelings of anxiety and depression), which in turn exert direct effects on biological processes or on behavioral patterns that influence disease risk (Cohen, Gianaros, & Manuck, 2016). Stress has been associated with a wide range of diseases and mortality (Cohen, Janicki-Deverts, & Miller, 2007; Cohen, Murphy, & Prather, 2019) and also with alterations in immune function with potential implications for increased risk for infectious diseases (Kiecolt-Glaser, McGuire, Robles, & Glaser, 2002).

### Psychological stress, colds, and influenza

In our first study of the role of psychological stress in susceptibility to upper respiratory disease (Cohen et al., 1991), we collected data from 394 healthy volunteers, using questionnaires to measure the number of recent major stressful life events (e.g., death of spouse, job loss), perceived stress (perception that demands on them exceed their ability to cope), and negative emotions (e.g., anxiety, depression). A stress index was defined as an equally weighed aggregate of these three indicators. Subsequently, we exposed each volunteer through nasal drops to one of five viruses (rhinovirus Types 2, 9, and 14; respiratory syncytial virus; or coronavirus 229E) that cause a mild common cold. We then followed them in quarantine for 6 days to determine whether they developed colds. Here we found that the higher the score on the stress index, the greater the likelihood that participants would develop a cold when later exposed to a virus. Those in the highest quartile were 2.16 times more likely to develop a cold than those in the lowest. Greater psychological stress was associated with greater risk for clinical illness in response to all five viruses and for both participants with (seropositive) and without (seronegative) earlier exposure to the virus (as indicated by detectable virus-specific antibodies at baseline). None of a group of 26 participants who received a placebo instead of a virus developed a cold.

In the next study, instead of stress questionnaires, we used an intensive life-event interview (the Life Events and Difficulties Schedule; Brown & Harris, 1989) that identified each individual’s most stressful life event, the type of event (e.g., interpersonal, educational, financial), and how long it lasted. After the interview, we exposed each of the 276 participants to one of two rhinoviruses (RV39 or Hanks) and monitored them in quarantine (Cohen et al., 1998). We found that the longer participants’ most stressful event lasted, the greater their probability of getting sick after virus exposure. Moreover, the types of events that were most predictive of colds were enduring interpersonal problems and being under- or unemployed. The relationships reported here were similar for the two virus types and for both those with and without previous exposure to the virus.

In the two psychological stress studies discussed above, we also tested whether stress predicted increased risk of disease because of its possible associations with elevated levels of “stress” hormones (epinephrine, norepinephrine, cortisol), poorer immune function (natural killer cell cytotoxicity, white blood cell counts), or risky behaviors such as smoking, poor diets, low levels of physical activity, and poor sleep, as well as the potential positive effects of low to moderate alcohol consumption (Cohen et al., 1997; Cohen et al., 1993). Contrary to expectations, none of these (alone or together) explained why stress was associated with greater risk of developing a cold.

New insights about the function of the immune system provided another possible mediator of the association between stress and disease risk. Chemicals called proinflammatory cytokines are released by the immune system in response to infections. These chemicals elicit an inflammatory response, drawing immune cells to the infected area to help orchestrate the immune defense against an infectious agent. Appropriate amounts of cytokine production facilitates the clearing of the virus. However, if the immune system produces too much of these inflammatory chemicals, the results can be toxic. In the case of infection with common cold and influenza viruses, producing too much proinflammatory cytokine triggers disease symptoms, such as nasal congestion and runny nose (Doyle, Skoner, & Gentile, 2005; Hayden et al., 1998; Short, Kroeze, Fouchier, & Kuiken, 2014).

In our next study (Cohen, Doyle, & Skoner, 1999), we examined the potential role of cytokine-produced inflammation in the link between stress and symptom production in response to an upper respiratory virus. We measured perceived stress by questionnaire and then exposed 55 participants to an influenza virus (influenza A/Kawasaki/86 H1N1). We measured how much local proinflammatory cytokine (interleukin-6) was found in participants’ nasal secretions on the day before virus exposure and on each of the 5 days after exposure. Participants who reported high levels of perceived stress at baseline produced higher levels of these inflammatory chemicals and in turn experienced more symptoms.

These results raised a dilemma for us. Acute stress exposures in the laboratory and natural settings have been found to increase circulating levels of cortisol (e.g., Cohen & Hamrick, 2003), a glucocorticoid hormone that normally reduces inflammation by suppressing the release of proinflammatory cytokines. Yet even though acute stress had been associated with increased cortisol, and hence would presumably decrease cytokine release, we found that people who suffered from chronic stress produced more, not less, proinflammatory cytokine (Cohen et al., 1999). In response to this apparent contradiction, we hypothesized that when people are exposed to major stressful events over a prolonged period, their bodies adapt to the initial increase in cortisol by reducing immune cell responsiveness to cortisol (a process called glucocorticoid resistance). As cells become less responsive, the body loses the ability to turn down the inflammatory response (Bailey, Engler, Hunzeker, & Sheridan, 2003).

We began testing this hypothesis by examining whether chronic stress in humans was associated with reduced responsiveness to cortisol. In a preliminary study (Miller, Cohen, & Ritchey, 2002), we identified a healthy population that was experiencing an intense and chronic stressful event, parents of children with cancer, and compared them with a matched group of relatively nonstressed parents of healthy children. There were 25 parents in each group. First, as expected, parents of patients with cancer reported more psychological stress than parents of healthy children. Second, when we added a synthetic cortisol-like glucocorticoid, dexamethasone, to blood samples from parents of healthy children, it reduced their immune cells’ ability to produce inflammatory chemicals. However, adding dexamethasone to blood samples from parents of patients with cancer was relatively ineffective in reducing the production of these chemicals. That is, immune cells from chronically stressed parents were insensitive to the regulatory effects of this cortisol-like glucocorticoid.

Finally, in two viral-challenge studies (Cohen et al., 2012), we tested the implications of chronic stress-elicited insensitivity to cortisol for susceptibility to disease. In both studies we exposed participants to one of two rhinoviruses (RV-21 and 39). In a study with 276 participants, we found that interpersonal stressful events lasting a month or longer were associated with a decrease in immune cells’ sensitivity to cortisol. In turn, decreased sensitivity to cortisol mediated the association between stressful life events and a higher risk of subsequently developing a cold. In a study of 72 participants, we found that lower sensitivity to cortisol was associated with greater production of proinflammatory cytokines in response to being infected by a cold virus.

In sum, the association between stress and disease occurs because chronic stress interferes with the body’s ability to turn off the immune system’s production of inflammatory chemicals; this failure in regulation (maintaining a proper level) of immune response occurs because chronic stress results in immune cells becoming insensitive to cortisol.

### Implications for COVID-19

We reported a series of studies demonstrating the association between psychological stress and increased risk for the common cold and influenza (also see reviews of broader literature in Cohen & Williamson, 1991; Pedersen, Zachariae, & Bovbjerg, 2010). These associations were found across eight cold and two influenza viruses and held irrespective of whether the participant had been previously exposed to the virus. In two cases, we found that this association was primarily mediated by greater rates of infection among those with higher stress levels. In the remaining studies, the association was primarily attributable to more symptoms among infected persons (which is possibly attributable to less statistical power in the later studies.) Overall, this suggests that stress may have a broad range of physiological effects relevant to responding to upper respiratory viruses.

Importantly, we found that chronic stressful events were associated with overproduction of proinflammatory cytokines and, in turn, with greater risk of illness and greater symptom scores. There is also evidence that increased cytokine levels correlate with disease severity of COVID-19 (Huang et al., 2020) and that continuous high levels are associated with disease deterioration and fatal outcomes. Moreover, preliminary evidence suggests that the disease may be attenuated by a cytokine antagonist (which blocks the action of cytokines by attaching to cytokine receptors; Luo et al., 2020; Nanda, 2020), to a kinase inhibitor (which blocks the activation of macrophages that produce inflammatory cytokines; Roschewski et al., 2020), and to dexamethasone (a glucocorticoid inhibitor of cytokine secretion; Ledford, 2020). Overall, these data suggest the possibility that chronic psychological stressors could play a role in the onset and progression of COVID-19 through their effects of cytokine regulation.

Another interesting aspect of our data on psychological stress and upper respiratory illness that has potential implications for the COVID-19 pandemic is the potential importance of interpersonal and economic stressors. These types of stressors may play a crucial role in the experience of prolonged sheltering in place that in turn could alter host resistance to the virus.

## Interpersonal Relationships and Disease Susceptibility

The nature and quality of our social relationships have proven to be important predictors of health and well-being (Cohen, 2004; Kiecolt-Glaser, Gouin, & Hantsoo, 2010; Robles, Slatcher, Trombello, & McGinn, 2014; Uchino, 2004). Two social-relationships concepts that have received considerable attention in predicting physical health outcomes are social integration and social support.

### Interpersonal relationships, colds, and influenza

Social integration refers to the degree to which an individual participates in a broad range of social relationships (Brissette, Cohen, & Seeman, 2000) and is generally defined in terms of the number of social roles (e.g., spouse, parent, friend, fellow employee, volunteer, church member) one plays. Social integration has been found to predict lesser mortality (reviewed by Holt-Lunstad, Smith, & Layton, 2010) as well as lower risk for cardiovascular disease onset (incidence) and disease progression (reviewed by Chin & Cohen, 2020). These associations are thought to occur because socially integrated people are subject to social pressures to take care of themselves and because integration is associated with positive psychological states (e.g., feelings of control, self-esteem, and positive affect) that have positive downstream effects on a range of disease-relevant physiological pathways (Cohen, 2004). In contrast, a particularly low level of integration is viewed as social isolation, which is experienced as a stressful event (Chin & Cohen, 2020).

After 276 healthy participants reported the extent of their participation in 12 types of social ties (e.g., spouse, parent, friend, workmate, member of social group), we exposed them to nasal drops containing one of two rhinoviruses (RV-39 or Hanks) and then monitored them for the development of a common cold (Cohen et al., 1997). Susceptibility to colds decreased with increased number of social roles. Those who were the least socially integrated (one to three social roles) were 4.2 times more likely to develop a cold than those who were most integrated (six or more). This association occurred in response to both viruses, occurred in both those with and without previous exposure, and was attributable to social integration, predicting both lower rates of infection and lower rates of illness among infected participants.

Note that the relationship between greater social integration and greater resistance to the virus was unaltered by statistical controls for baseline (before challenge) virus-specific antibody. That is, it could not be explained by the idea that more socially integrated people were resistant to virus-induced illness because their diverse networks resulted in a greater probability of earlier exposure to the virus that in turn resulted in immunity to the virus. Although smoking, poor sleep quality, alcohol abstinence, and low dietary intake of vitamin C were all associated with greater susceptibility to colds (see above), they could account for only a small proportion of the relation between social integration and decreased cold risk.

Social support refers to the resources provided by one’s social network in the face of adversity. Perceived social support has been hypothesized to protect against the pathogenic effects of stress (Cohen, 2004; Cohen & Wills, 1985). This is usually attributed to the belief that others would help in the face of adversity by reducing one’s appraisal of a situation as threatening (Cohen, 2004). Using a sample of 404 healthy adults, we examined whether perceived social support buffered the increased risk for colds found among those suffering from enduring interpersonal stressors (Cohen, Janicki-Deverts, Turner, & Doyle, 2015). As discussed earlier, social conflicts were found to be associated with greater risk of colds among persons exposed to a cold virus (Cohen et al., 1998). We assessed perceived social support at baseline (before viral challenge) using a validated questionnaire, and we assessed daily interpersonal conflicts by telephone interviews on 14 consecutive evenings. Subsequently, participants were exposed to a rhinovirus (RV-39) or an influenza virus (A/Texas/36/91) and were monitored in quarantine to assess infection and illness signs. Perceived support protected against the increased risk for developing an infection associated with a greater percentage of conflicts over the 14-day baseline, and this association held for both viruses.

### Implications for COVID-19

Social integration has been associated with a decrease in the risk for multiple diseases and death. Our work indicated that greater social integration was similarly associated with decreased risk of upper respiratory illness for persons exposed to two different rhinoviruses, irrespective of whether they had previous exposure to the viruses. Moreover, the association of social integration and colds was attributable to decreases in both infection and illness expression among infected participants, allowing for multiple pathways through which integration may influence response to SARS-CoV-2. Because integration effects are sometimes driven by the detrimental effects of the lowest levels of integration (Chin & Cohen, 2020), it is reasonable to hypothesize that, for some, the social isolation inherent in sheltering at home may contribute to greater susceptibility to infection and disease if they are exposed to the virus.

As discussed earlier, chronic interpersonal stressors were associated with greater risk for illness among persons we exposed to a virus. In our work with both a cold virus (rhinovirus) and an influenza virus, we found that support perceptions were protective in the face of ongoing interpersonal stress. These data are consistent with other work on the protective role of social support, including studies of psychological and physical health outcomes (Cohen, 2004; Cohen & Wills, 1985). Specifically, it suggests the possibility that perceived support may operate as a resilience factor for stressed persons exposed to SARS-CoV-2. In the face of the pandemic-triggered quarantine, support perceptions may be of particular importance in coping with the isolation and/or intense interaction with family associated with sheltering at home.

## Discussion and Implications for COVID-19

The common cold is an infection of the upper respiratory tract but (at least in response to rhinovirus) may produce lower airway dysfunction and trigger asthma exacerbations. Influenza is also primarily upper respiratory, but the virus and its products can also concentrate in the lungs. Although SARS-CoV-2 is found in the upper respiratory system, it appears to primarily concentrate in the lungs. However, the concentration of the virus varies over the course of the disease.

At the simplest level, the common cold, influenza, and COVID-19 have some overlapping symptoms, including sore throat, cough, headache, tiredness, and, in the case of influenza and COVID-19, muscle pain, chills, and fever (Huang et al., 2020). Those with severe cases of influenza (Short et al., 2014) or COVID-19 (Baas, Taubenberger, Chong, Chui, & Katze, 2006) may experience pneumonia and/or acute respiratory distress syndrome, in which fluid builds up in the tiny, elastic air sacs (alveoli) in the lungs. The fluid keeps the lungs from filling with enough air, making it difficult to breathe and preventing oxygen from reaching the bloodstream.

There are other reasons to think that our results may provide a map for investigating the role of psychosocial factors in susceptibility to COVID-19 among exposed individuals. First, the associations between psychosocial factors and upper respiratory illnesses that we have reported are similar across the upper respiratory viruses. We used a total of eight common cold (rhinovirus Types 2, 9, 14, 21, 39 and Hanks; respiratory syncytial virus; and coronavirus 229E) and 2 influenza viruses (A/Kawasaki/86 H1N1; and A/Texas/36/91), and data were always consistent across viruses within and between studies. For example, the association between psychological stress and colds was found with all 10 of the viruses. Notably, one of the cold viruses employed in our early research on psychological stress and on smoking and alcohol consumption is one of the four coronaviruses (CoV 229E) that cause colds. Overall, this suggests the existence of common pathways linking psychosocial factors to disease among a wide range of respiratory viruses.

A strong point for the argument that our work may enlighten us about risk for COVID-19 infection and disease derives from the important role of proinflammatory cytokines in colds, influenza, and COVID-19. In both the common cold (Doyle et al., 2005) and influenza (Hayden et al., 1998; Short et al., 2014), poor regulation of proinflammatory cytokine response to the viral infection (i.e., too much proinflammatory cytokine) triggers the production of respiratory symptoms. A similar process seems to be at play in response to SARS-CoV-2 (Nanda, 2020; Roschewski et al., 2020; Yang et al., 2020). A coordinated and controlled cytokine response is essential for an effective immune response to SARS-CoV-2. However, as many as 15% of those battling a serious infection fail to turn off proinflammatory cytokine production (referred to as a *cytokine storm*), resulting in excessive inflammation and in turn damage to multiple organs, including the lungs and liver, that may eventually lead to death (Baas et al., 2006; Yang et al., 2020).

Excessive inflammation is thought to mediate the associations with upper respiratory disease for nearly all of the disease predictors in the work I have presented. Our own data indicate that the association between chronic psychological stress and disease is mediated by poor proinflammatory cytokine regulation (i.e., too much cytokine), and work by others has suggested that excessive inflammation may mediate (or partially mediate) the associations between the expression and severity of symptoms of respiratory infections and smoking (Zhou et al., 2016), alcohol consumption (Ridker et al., 1998), poor sleep (Irwin, 2015), and insufficient exercise (Martin et al., 2009; Nieman, 1994). Although inflammation has not been investigated as a mediator of interpersonal relationships and respiratory illness, social integration and support have both been found to be associated with lower circulating levels of inflammatory markers (see meta-analysis by Uchino et al., 2018). Thus, the psychosocial risk factors discussed in this article as predictors of colds and influenza may play a similar role in COVID-19, another respiratory disease in which poor regulation of proinflammatory cytokine response is thought to drive disease, especially in the most severe cases.

Our studies have some limitations in term of their similarities to data from the pandemic. Early evidence suggests that deaths and serious illness from COVID-19 are concentrated among those who are older and who have underlying health issues, such as diabetes, cancer, and respiratory conditions. Our samples are limited to quite healthy and relatively young (18–55 years old) individuals, and hence it is difficult to estimate whether the patterns of results we find would generalize to the most seriously ill COVID-19 patients. COVID-19 also has a broader array of symptoms than do colds and influenza (e.g., loss of taste and smell, and blood clotting), which suggest that other disease mechanisms are also in play.

## Final Comments

We have summarized the results from three of our areas of research on psychosocial factors and susceptibility to upper respiratory viruses (see Table 2; also see the CCP website for other factors that may influence susceptibility). As noted earlier, I hope that the integrated presentation of this research will inform both the public and scientific community of the importance of behavior, psychological states, and social interactions to our health and, in particular, to the onset and progression of respiratory infectious diseases.

**Table 2.** Summary of Psychosocial Factors Associated With Risk for Upper Respiratory Infectious Disease Among Those Exposed to a Virus

Psychosocial factor	Association with upper respiratory disease
Health-related behaviors	
Smoking	Greater risk
Alcohol consumption	Moderate drinking incurs less risk
Exercise	Lack of minimum exercise (2 days/week) at greater risk
Vitamin C	Less than daily requirement (85 g) at greater risk
Sleep	Fewer than 6–7 hr a night at greater risk
	Lower sleep efficiency at greater risk
Psychological stress	
Aggregate measure	Increased stress associated with increased risk
Perceived stress	Increased stress associated with increased risk
Severe stressful event	The longer the event lasts, the greater the risk
	Prolonged interpersonal and economic events are the most potent
Interpersonal	
Social integration	The more social roles, the lesser the risk
Social support	The greater the perceived support, the lesser the risk for high-stressed but not for low-stressed persons (stress-buffering)

In relation to the current pandemic, I also have presented arguments suggesting that these data can help direct the science necessary to identify which people exposed to SARS-CoV-2 are at risk for infection, disease onset, and disease progression. In turn, this should help the public and public-health administrators to identify what constitutes high risk for those exposed to the virus and focus interventions appropriately. The argument that work from the viral-challenge trials are relevant here is based on similarities among the symptoms of colds, influenza, and COVID-19 and in the role of proinflammatory cytokine regulation in disease pathogenesis, as well as on the consistency of psychosocial factors as predictors of disease susceptibility across multiple respiratory viruses. The hypotheses for the generality of each factor discussed here are made with caution, because although there are similarities among colds, influenza, and COVID-19, there are also major differences (Paules, Marston, & Fauci, 2020).

Do our data tell us anything specifically about the potential effects of the COVID-19 pandemic? It is generally accepted that sheltering at home, quarantine for ill patients, and job loss can trigger psychological distress, anxiety, and depression (reviewed by Brooks et al., 2020) and that strong support networks may attenuate these effects (Centers for Disease Control and Prevention, 2019). Our work suggests that chronic interpersonal and employment-related stressors are also potent risks for upper respiratory disease for those exposed to respiratory viruses and that social integration and social support may confer resilience. Diverse networks and strong support systems may be especially beneficial to the extent that network contact can be maintained by phone, social media, Zoom, FaceTime, and so forth (Hobbs, Burke, Christakis, & Fowler, 2016). It is also possible that those with low levels of support and social integration suffer more in quarantine because of a lack of contacts to engage when isolated. Overall, the evidence from the viral-challenge trials indicates that the experiences associated with the quarantine (including sheltering at home), its potential for interpersonal stressors (including isolation, loneliness and conflict), and the loss of employment are particularly powerful predictors of host resistance to respiratory viruses. In turn, it is possible that they might play a similar role in susceptibility to COVID-19.

## Transparency

*Action Editor:* Laura A. King

*Editor:* Laura A. King
